# Spatial and temporal variations of air pollution over 41 cities of India during the COVID-19 lockdown period

**DOI:** 10.1038/s41598-020-72271-5

**Published:** 2020-10-06

**Authors:** Krishna Prasad Vadrevu, Aditya Eaturu, Sumalika Biswas, Kristofer Lasko, Saroj Sahu, J. K. Garg, Chris Justice

**Affiliations:** 1 NASA Marshall Space Flight Center, Huntsville, AL 35811 USA; 2 University of Alabama in Huntsville, Huntsville, AL USA; 3 Smithsonian Conservation Biology Institute, Front Royal, VA USA; 4 US Army Corps of Engineers, Alexandria, VA USA; 5 Utkal University, Bhubaneswar, Odisha India; 6 Tata Energy Research Institute (TERI) School of Advanced Studies, New Delhi, India; 7 University of Maryland, College Park, MD USA

**Keywords:** Environmental impact, Environmental sciences

## Abstract

In this study, we characterize the impacts of COVID-19 on air pollution using NO_2_ and Aerosol Optical Depth (AOD) from TROPOMI and MODIS satellite datasets for 41 cities in India. Specifically, our results suggested a 13% NO_2_ reduction during the lockdown (March 25–May 3rd, 2020) compared to the pre-lockdown (January 1st–March 24th, 2020) period. Also, a 19% reduction in NO_2_ was observed during the 2020-lockdown as compared to the same period during 2019. The top cities where NO_2_ reduction occurred were New Delhi (61.74%), Delhi (60.37%), Bangalore (48.25%), Ahmedabad (46.20%), Nagpur (46.13%), Gandhinagar (45.64) and Mumbai (43.08%) with less reduction in coastal cities. The temporal analysis revealed a progressive decrease in NO_2_ for all seven cities during the 2020 lockdown period. Results also suggested spatial differences, i.e., as the distance from the city center increased, the NO_2_ levels decreased exponentially. In contrast, to the decreased NO_2_ observed for most of the cities, we observed an increase in NO_2_ for cities in Northeast India during the 2020 lockdown period and attribute it to vegetation fires. The NO_2_ temporal patterns matched the AOD signal; however, the correlations were poor. Overall, our results highlight COVID-19 impacts on NO_2_, and the results can inform pollution mitigation efforts across different cities of India.

## Introduction

In early 2020, the COVID-19 virus started to spread rapidly across the globe into most countries, including India where the first case reported on January 30th, 2020. The latest information pertaining to the number of COVID-19 active cases, cured discharged statistics, and other public health-related information is reported on the Ministry of Health and Family Welfare, Government of India website (https://www.mohfw.gov.in/). As per the website on May 20th, 2020, the total number of active cases was reported to be 61,149, with 42,297 cured/discharged and 3,303 deaths. Of the different states, Maharashtra had the highest number of cases, followed by Gujarat, Delhi, Tamil Nadu. There are currently no confirmed cases reported in Arunachal Pradesh, Sikkim, Nagaland, Mizoram, etc.

### India lockdown period

With the COVID-19 outbreak spreading in more than twelve states, by the third week of March 2020, the Government of India invoked the Epidemic Diseases Act, 1897 and government, educational, commercial establishments were shut down, including the suspension of all tourist visas. Initially, on March 22nd, 2020, the Prime Minister announced a 14-h public curfew for the country, with lockdowns in seventy-five districts where COVID-19 cases had occurred. Shortly thereafter, on March 24th, a nationwide Phase-1 lockdown was announced for 21 days (March25–April 14th, 2020) affecting the entire 1.3 billion population of India.

Further, on April 14th 2020, the Prime Minister implemented Phase-2 which extended the ongoing nationwide lockdown until May 3rd. During the lockdown, all commercial and non-commercial activities came to a halt. For example, all factories, markets, and shops were closed, including any public gathering and places of worship. People across the country were asked to stay home and practice social distancing if they could not remain at home. A recent report by the Oxford COVID-19 Government Response Tracker^1^, based on data from 73 countries reported that India had one of the most stringent measures with respect to “swift action, emergency policy-making, emergency investment in healthcare, fiscal measures, investment in vaccine research and active response to the situation, and scored India with a “100” for its strictness”^1^.

After nearly five weeks of total nationwide lockdown, the Phase-3 of the lockdown was announced from May 4th–May 17th 2020, characterized by partial reopening. The 733 districts in the country were divided into green, orange, and red zones based on the number of active COVID-19 cases. The green and orange zones were given a relatively relaxed set of restrictions, whereas the red zones were more restrictive. For example, in a red-zone, entry and exit was restricted with specific timings to obtain grocery essentials. Also, in the red-zone, e-commerce players could not deliver non-essentials, and only permitted bicycle, autorickshaws, and taxicabs traffic. Private establishments were allowed to operate with a 33% staff strength, and movement was not permitted between 7 pm and 7am.

Phase 4 of the nationwide lockdown was announced on May 17th, 2020 and extended until May 31st. As a part of the fourth phase, the States and Union Territories (UT’s) of India were given the authority to delineate Red, Green, and Orange Zones as a function of how the COVID-19 situation evolved. The fourth phase instituted a slow reopening with several relaxations. For example (a) inter-state movement of passenger vehicles was permitted with mutual consent between states; the intra-state movement of passenger vehicles and buses, to be determined by States and UTs; (b) essential services were allowed to resume within specified containment zones; (c) restaurants were permitted to operate kitchens only for home delivery of food, etc. (d) sports complexes and stadiums were allowed to open, without spectators. Overall, the Government of India has been following stringent measures to reduce the spread of COVID-19. Of the four different phases, the most restrictive lockdown phase was from March 25th–May 8th, 2020 (Phase-1 and 2) which is the focus of this study.

## Questions addressed

It is well known that air pollution in several regions of the world is due largely to human activities, such as from fossil fuel combustion from motor vehicles, industries, power plants, etc. With the COVID-19 pandemic, a reduction in pollution has been reported by several researchers in different regions of the world such as Italy, the USA and Spain^2–5^. As a result of the COVID-19 pandemic, during March 25th–May 3rd, 2020 entire India was lockdown. As a result, there was reduction in pollution in Indian cities too^6,7^. However, the specific amount of pollution decrease is not well-documented covering multiple cities in India, hence the focus of this study. Some of the metropolitan cities such as New Delhi, Bangalore, Mumbai in India are renowned for its air pollution. Since, cities are hotspots of air pollution, we focused on 41 cities based on their population size and analyzed how the air pollution varied during the lockdown period as compared to the previous year as well as the pre-lockdown period. We addressed the following questions: (a) How much was NO_2_ pollution reduced during Phase-1 and 2 of the COVID-19 full country lockdown (March 25-May 3rd, denoted here as 2020-lockdown)? (b) Specifically, how did NO_2_ in the 2020-lockdown compare to the same period in 2019, when there was no lockdown (denoted as 2019-no lockdown)? (c) How did NO_2_ levels during the 2020-lockdown compare with January–March 24th 2020 (denoted here as 2020-pre-lockdown)? (d) Were the differences in NO_2_ pollution reduction consistent across 41 cities? (e) Which cities had the highest and least reduction in NO_2_? (f) Are there scaling effects in NO_2_ levels in cities, i.e., based on the spatial distance to the city center? (g) What was the overall reduction in NO_2_ for major cities across India and are the differences statistically significant? We addressed these questions using the remote sensing derived TROPOMI-NO_2_ datasets and the MODIS Aerosol Optical Depth (AOD) data covering different cities in India. We focused on satellite-derived NO_2_ only since the measurement algorithm is relative matured ones compared to the other gases. Also, adverse health effects of NO_2_ include acute respiratory illness, decreased pulmonary function, asthma, lung cancer and cardiopulmonary mortality^8^; thus, it is important to address spatial and temporal variations in NO_2_ useful for pollution management and mitigation purposes.

## Cities studied

**Figure 1:**
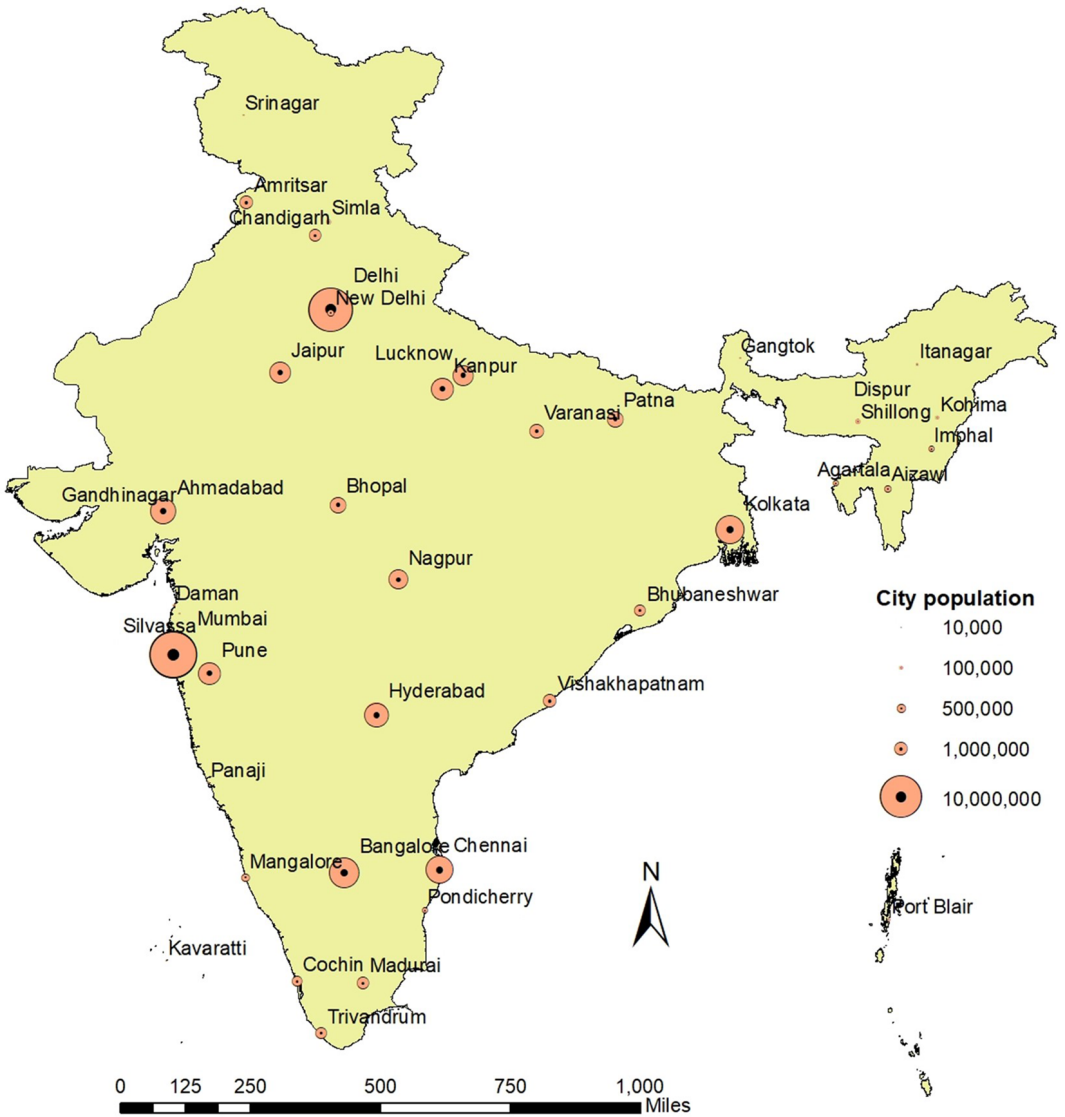
Map of India showing location and population in 41 different cities (QGIS software (3.10) QGIS.org (2020) was used, accessible from https://qgis.org/).

We selected the 41 cities in India, based on 7 different categories ranked by population (Fig. 1). Rank-1 cities have the highest population of 5.0 million or greater, and rank-7 cities have less than 50,000 people. A map of the 41 cities selected for the study is shown in Fig. 1. The results of our analysis of the spatial and temporal variation in the pollution levels are presented for: (a) individual cities; (b) averaged results based on the city’s population ranking; (c) the top-seven highest polluted cities; (e) cities in northeast India and (f) coastal cities.

## Datasets

We used the TROPOspheric Monitoring Instrument (TROPOMI) onboard the Sentinel 5 precursor (S5P), operated by the European Space Agency (ESA)^9^ to assess the tropospheric NO_2_ background levels. Sentinel-5 Precursor (Sentinel-5 P), launched on 13th October 2017, was the first Copernicus mission satellite and can measure several trace gases such as NO_2_, ozone, formaldehyde, SO_2_, methane, carbon monoxide, and aerosols. The resolution is for all gases with 3.5 × 7 km^2^, except for CO and CH_4_, which is 7 × 7 km^2^. The TROPOMI instrument contains three spectrometers that cover the ultraviolet-near infrared region with two spectral bands at 270–500 nm and 675–775 nm and one spectrometer that covers the shortwave infrared band. Relatively, TROPOMI has a higher resolution compared to its predecessor, OMI which has a ground resolution of 13 km × 24 km at nadir. The TROPOMI NO_2_ retrieval algorithm utilizes the bands of the ultraviolet-near-infrared spectrometer^10^. The retrievals are based on the NO_2_ DOMINO system which was previously used for OMI spectra^11^ with additional improvements^10^. The NO_2_ slant column density is retrieved using the differential optical absorption spectroscopy (DOAS) method and separated into stratospheric and tropospheric components using the information from the data assimilation system and separation based on the altitude dependent air mass factors based on the lookup table approach. The final product provides the tropospheric vertical column densities, which describes the vertically integrated number of NO_2_ molecules per unit area from the surface to tropopause. The data can be accessed either through the Near-Real-Time (NRTI) stream, the Offline stream (OFFL), or the Reprocessing (RPRO) stream. NRTI data are available within three hours after data acquisition, whereas OFFL and RPROdata are available within a few days after acquisition^10^. In this study, we used the TROPOMI, near-real-time operational product^9^ obtained via the Copernicus open data access hub (https://s5phub.copernicus.eu). Independent validation by the S5P Mission Performance Center (MPC) and S5P validation team concluded that OFFL level 2 NO_2_data are in overall agreement with reference measurements collected from global ground-based networks^12–14^. In addition to TROPOMI NO_2_, we also used the MODIS product MCD19A2.006: Terra and Aqua Multi-angle Implementation of Atmospheric Correction (MAIAC) Land Aerosol Optical Depth (AOD) gridded Level 2 product, specifically, the blue band (0.17um) 1-km daily data over land^14,15^ for our study. All processing was done using the QGIS software (3.10) “QGIS.org (2020). QGIS Geographic Information System. Open Source Geospatial Foundation Project https://qgis.org/”.

## Methods

To generate time series of NO_2_ columns over 41 different cities, we first selected pixels from an overpass area, defined by a different buffer radius (30, 45, 60, 75, 90 in km from the city center). We used data for which the quality assurance value is higher than 0.5 and the cloud fraction within the NO_2_ retrieval window is below 40%^6^. The averaged tropospheric NO_2_ column for each city within the buffer radius is calculated as,

$${A}_{k, d}=\frac{{\sum }_{k=1}^{kmax}{\mathrm{D}}_{\mathrm{k},\mathrm{ d}}}{{\sum }_{k=1}^{kmax}{\mathrm{N}}_{\mathrm{k},\mathrm{ d}}}$$

where, ${A}_{k, d}$ is the average value of the data for each city during the time period of observations, ${D}_{k, d}$ is the average value of the data for each grid cell ‘k’ within the buffer radius (in km), for each day ‘d’ of the month, over the time period of the observations and ‘${\mathrm{N}}_{\mathrm{k},\mathrm{ d}}$’ being the total number of days of observations for each city within a specific period, i.e., before or after lockdown. After obtaining the averaged NO_2_ value for individual cities (${A}_{k, d}$) within a specified buffer distance and time period, we then used the individual values for all the 41 cities to obtain an average for entire India as,

$${M}_{d,c}=\frac{{\sum }_{k=1}^{kmax}{(\mathrm{D}}_{\mathrm{k},\mathrm{ d},\mathrm{ c}}*{N}_{k, d, c }) }{{\sum }_{k=1}^{kmax}{\mathrm{N}}_{\mathrm{k},\mathrm{ d},\mathrm{ c}}}$$

where, ‘${M}_{d,c}$’ is the average NO_2_ for 41 cities during the period of observations i.e., before and after lockdown, ${\mathrm{D}}_{\mathrm{k},\mathrm{ d},\mathrm{ c}}$ is the average NO_2_ for each city over a period of observations with each grid cell within the city as ‘k’, day ‘d’ and with ${N}_{k, d, c}$ being the total number of days of observations for all cities.

### Paired t-test

We used the paired t-test^16,17^ to compare the mean differences between NO_2_ pollution levels during different months for the previous (2019) and the current year (2020). The t-test follows a Student's t-distribution under the null hypothesis of H0 that the means are equal, H_0_: µ_1_ = µ_2_ with the alternative hypothesis that H_a_: µ_1_ ≠ µ_2_. The p-value is used to reject or accept the null hypothesis. The H_0_ hypothesis was discarded when the p-value was less than 0.05 (significance level of 5% in this study) and the H_a_ hypothesis is accepted^18^.

### Autoregressive moving average model with intervention

We used the univariate autoregressive moving-average (ARMA) analysis^19,20^ with the intervention^21,22^ to quantify the impacts of COVID-19 on the pollution levels. Specifically, ARMA models are developed as linear functions of NO_2_ values with the random shocks or errors based on the lockdown dates. The main difference between the ARMA model and ARIMA model is the integral part of the latter, i.e., a measure of how many nonseasonal difference values are needed to obtain stationarity. Thus, if no differencing is involved, then the model becomes ARMA. In this study, we implemented the ARMA modeling framework in three important steps^8^ (a) identification of the model; (b) estimation of the coefficients and (c) verification of the model. All these steps are implemented in an iterative process, resulting in a number of tentative models. First or second-order differencing (nonseasonal and/or seasonal) is useful for the non-stationary means. The identification of the number of terms to be included in the ARMA model is based on the analysis of the autocorrelation (ACF) and partial autocorrelation (PACF) functions of the differenced time series data. The model coefficients were estimated by means of the maximum likelihood method. Also, the verification of the model is performed through diagnostic checks of residuals through the normal probability plots and standardized residuals. Finally, Akaike’s Information Criteria (AIC) and Log-likelihood criterion were used to establish the model fit.

The intervention analysis helps to determine whether an event affects a timeseries of data, the known source and timing of intervention due to COVID-19, and the datasets in our case are is 2020-pre lockdown (January 1st–March 24th, 2020) versus 2020 lockdown period (March 25th–May 8th, 2020). The basic ARIMA model is given as (Eq. 1), when the intervention-free time series Zt­follows the ARIMA (p = autoregressive parameter or the number of lag observations included in the model, also called the lag-order; d = the number of times the raw observations are differenced or degree of differencing and q = size of the moving average window) × (P,D,Q)s (pre-intervention) model with the seasonal period of S, an external shock, mt, has an additive impact. Zt is the time series before the COVID outbreak and mt is the function indicating the impact of the outbreak.

$${Y}_{t}={m}_{t}+{Z}_{t}$$

1 $${\left(1-B\right)}^{d}{\left(1-B\right)}^{D}{\Phi}_{p}\left(B\right){\Phi}_{p}\left({B}^{s}\right){Z}_{t}= {\theta}_{0}+{\theta}_{q}\left(B\right){\Theta }_{Q}\left({B}^{s}\right){a}_{t}$$

In the above equation, Y_t_ includes the intervention, ${\Phi}_{p}\left(B\right)$ is a non-seasonal AR polynomial, ${\Phi}_{p}\left({B}^{s}\right)$ is a seasonal AR polynomial, ${\theta}_{q}\left(B\right)$ is the non-seasonal MA polynomial, ${\Theta }_{Q}\left({B}^{s}\right)$ is the seasonal MA polynomial, and a_t_ is the white noise WN (0, σ^2^). As mentioned, we used only ARMA model in our study.

Further, following the Box and Jenkins^19^, the intervention effect m_t_ (due to COVID-19 in our case) can be calculated as either with the pulse function ${P}_{t}^{(T)}$ or the step function ${S}_{t}^{(T)}$.

2 $${P}_{t}^{(T)}=\left\{\begin{array}{c}0, t\ne T\\ 1, t=T\end{array}\right.$$

3 $${S}_{t}^{(T)}=\left\{\begin{array}{c}0, t<T\\ 1, t\ge T\end{array}\right.$$

The pulse function is generally used when a certain event happens at time T, and its effect is limited (Eq. 2), whereas step function is used when the event is continuous after T (Eq. 3). In the above calculation, an indicator function either a unit step or a unit pulse^20^ are transformed by an AR(1) process with a parameter delta, and then scaled by a magnitude which is the coefficient on the transformed indicator function^21,22^. Thus, the model can represent changes that are abrupt and permanent (step function with delta = 0, or pulse with delta = 1), abrupt and non-permanent (pulse with delta < 1), or gradual and permanent (step with delta < 0). The algorithm is based on the ARMA transformation and linear regression to find the magnitude. We tried a step function, as our data fits such a context (with COVID-19 impacts on NO_2_ reduction, which continued from March 25th to April 3rd) to arrive at the smallest standard error on the magnitude^22^ with the ARMA intervention analysis^23^. The ARMA results are reported for before and after the intervention for seven dominant cities where NO2 pollution was most evident.

## Results

### NO_2_ variations for all 41 cities

**Figure 2:**
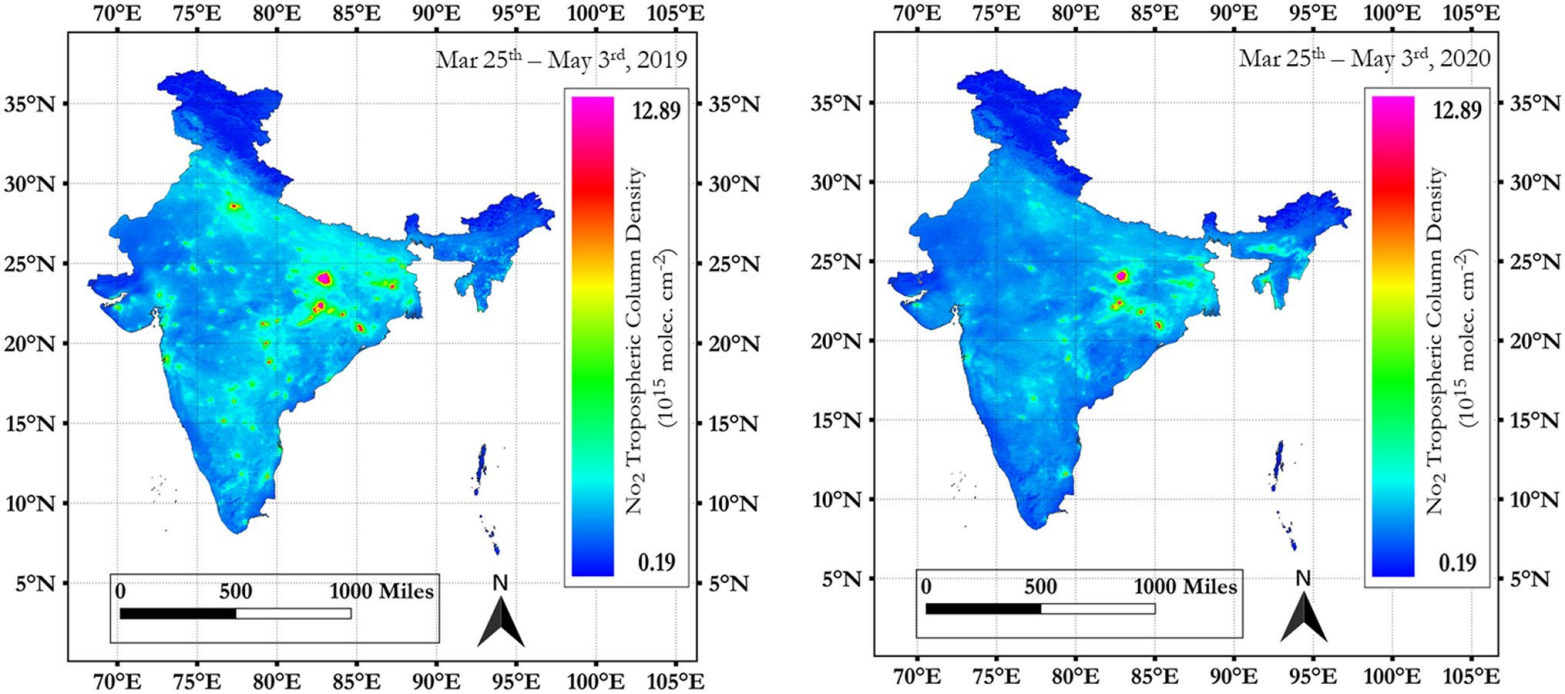
Spatial variations in mean tropospheric NO_2_ during 2019 (March 25th–May 3rd) non lock down versus 2020 (March 25th–May 3rd) COVID lock down period, India (QGIS software (3.10) QGIS.org (2020) was used, accessible from https://qgis.org/).

**Figure 3:**
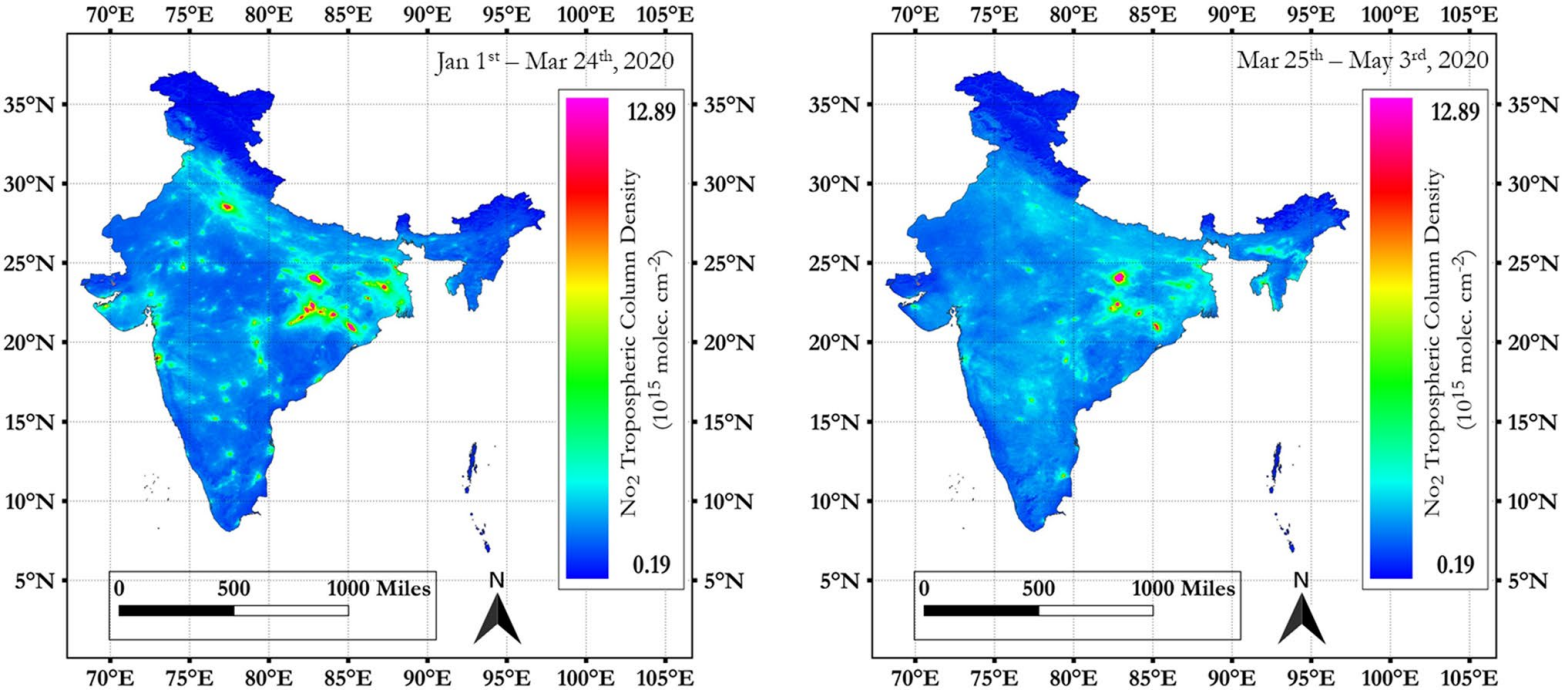
Spatial variations in mean tropospheric NO_2_ during 2020 (January 1st–March 24th) non lock down versus 2020 (March 25th–May 3rd) COVID lock down period, India (QGIS software (3.10) QGIS.org (2020) was used, accessible from https://qgis.org/).

Spatial variations in mean tropospheric NO_2_ during 2019 (March 25th–May 3rd) non-lockdown versus 2020 (March 25th–May 3rd) COVID lockdown period for India is shown in Fig. 2 and 2020 (January 1st–March 24th) pre-lock down versus 2020 COVID lockdown period, is shown in Fig. 3. Also, details for each city for the mean tropospheric NO_2_ variations for 2020 pre and post-lockdown periods is provided in Supplementary Materials.

**Figure 4:**
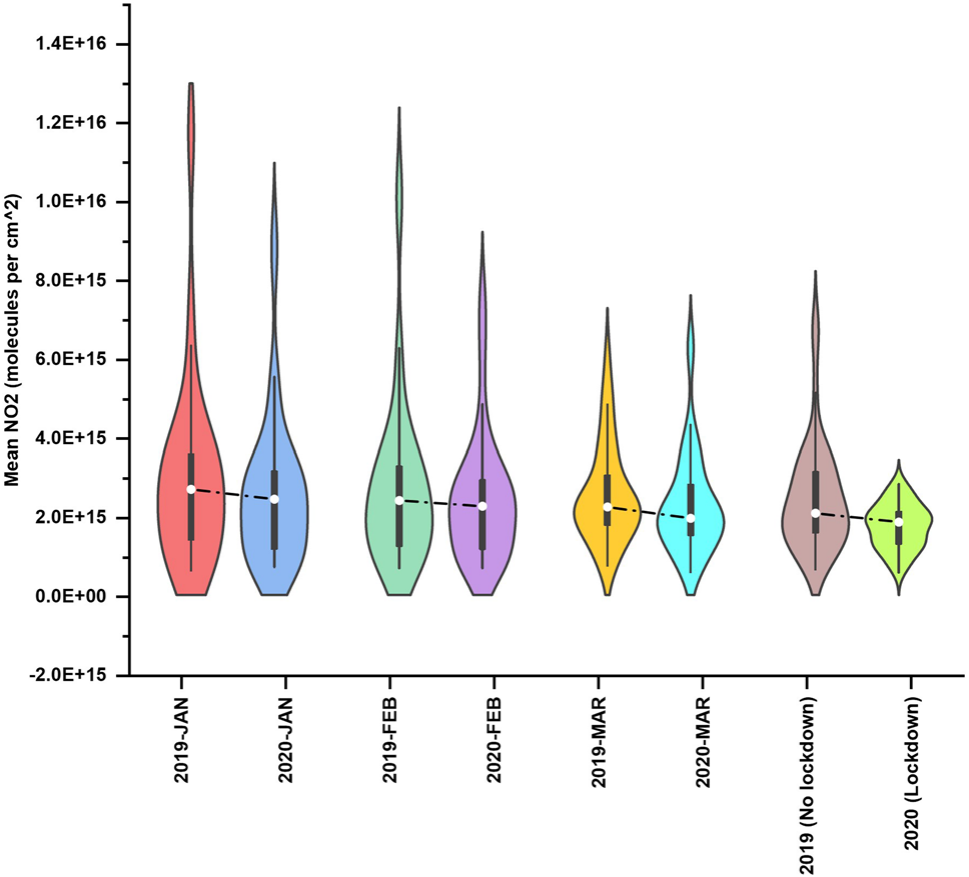
Violin plot depicting NO_2_ variations for 41 cities in India. A clear reduction in NO_2_ can be seen during the 2020 lock down period (March 25th–May 3rd, 2020).

To infer the data quality, we used the violin plots (Fig. 4) that combine the basic summary statistics of a box plot with a kernel density plot. In the violin plots, the thick black bar in the center represents the interquartile range, the central white dot represents the median value, and the whiskers show a 1.5 × interquartile range (IQR) in the rest of the data. On each side of the black line is a kernel density estimation to show the shape of the data distribution. The wider sections of the violin plot represent a higher density of observations, and the skinnier sections represent lower density. Thus, for example, for the 2020 lockdown data, the violin is thicker in the center, suggesting that most of the values had consistently higher frequency around the median. In contrast, 2019-January and 2019-February data had relatively higher tapering ends with elongated distribution of the data compared to the other plot. Further, a clear decrease in the median NO_2_ value can be seen, i.e., in general, the NO_2_ pollution during the 2020 pre-lockdown period was considerably less for all 41 cities compared to 2019. The IQR is a measure of variability; thus, for 2019-January data, it is higher, suggesting the NO_2_ values are more spread out from the median value compared to the 2020 lockdown period (Fig. 4). While we don’t intend to quantify drivers of these variations, there are many complex interacting factors like transportation, industry, biomass burning, etc., that might have affected these values.

The paired t-test was quite useful to infer the statistical significance between the two datasets for different months of 2019-no lockdown versus 2020 lockdown. For example, the results from January-2019 versus January-2020 mean tropospheric NO_2_ for all 41 cities suggested an overall reduction by 11%, and the results from the paired t-test were statistically different (January 2019, M = 3.05e+15, SD = 2.53e+15) versus (January 2020, M = 2.57e+15, SD = 1.90e+15); t(40) = (4.21), p = 0.0001. Since the p-value is less than 0.05 (significant level of 95%), we rejected the null hypothesis and accepted the alternate hypothesis that mean differences between the two independent data exists, suggesting a decrease in pollution.

The results from February-2019 NO_2_ versus February-2020 NO_2_ suggested an overall reduction of 8%, and the results from the paired t-test were statistically different (February 2019, M = 2.75e+15, SD = 2.13e+15) versus (February 2020, M = 2.43e+15, SD = 1.63e+15); t(40) = (2.992), p = 0.0047. Since the p-value is less than 0.05 (significant level of 95%), we rejected the null hypothesis and accepted the alternate hypothesis that mean differences between the two independent data exists, suggesting a decrease in pollution.

Similarly, analysis for March-2019 NO_2_ versus March 24th 2020 (pre lockdown) NO_2_ suggested an overall NO_2_ reduction of 12% and similar to January and February, the results were statistically different (March-2019, M = 2.55e+15, SD = 1.97e+14) versus (March-2020, M = 2.28e+15, SD = 2.0e+14); t(40) = (4.940), p = 0.000. Since the p-value is less than 0.05 (significant level of 95%), we rejected the null hypothesis and accepted the alternate hypothesis that mean differences between the two independent data exists, suggesting a decrease in pollution.

Analysis of data between March 25th to May 2019 (no-lockdown period) versus March 25th to May 3rd 2020 (COVID lockdown period) suggested an overall NO_2_ reduction of 19% and the results are statistically different (2019 no lockdown, M = 2.45e+15, SD = 1.38e+15) versus (2020 lockdown, M = 1.74e+15, SD = 5.74e+14); t(40) = (4.393)p = 0.0001. Since the p-value is less than 0.05, we rejected the null hypothesis and accepted the alternate hypothesis that mean differences between the two independent data exists. In summary, these results clearly suggest a statistically significant reduction in NO_2_ pollution during the 2020 for different months and the lockdown period.

### Top seven cities with highest NO_2_ pollution reduction

**Figure 5:**
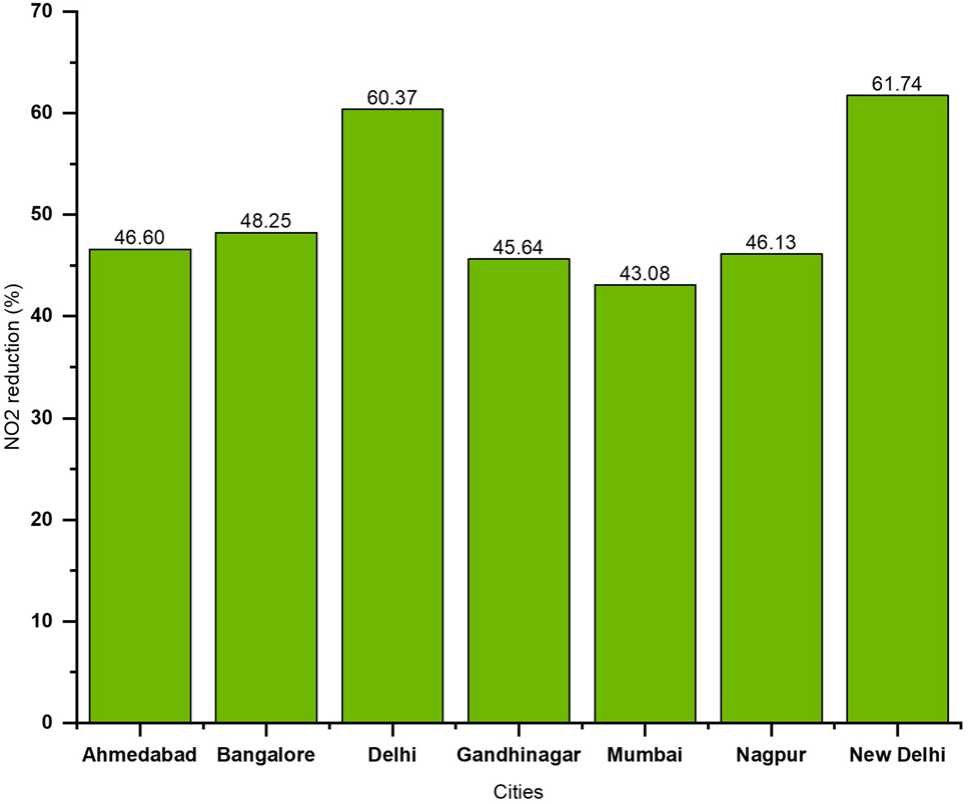
Top seven cities in India with NO_2_ reduction during the lockdown period (March 25th–May 3rd, 2020).

The top-seven cities with the highest NO_2_ pollution reduction based on the data from 2019 no-lockdown versus 2020 COVID lockdown period at 30 km radius from the center were New Delhi (61.74%), Delhi (60.37%), Bangalore (48.25%), Ahmedabad (46.20%), etc. (Fig. 5). The mean reduction of NO_2_ in these seven cities during the 2020 lockdown period is 50.27%. Further, we also calculated the NO_2_ variations during the 2020-pre lockdown versus 2020 lockdown and found an almost 50.47% reduction during the 2020 lockdown period.

**Figure 6:**
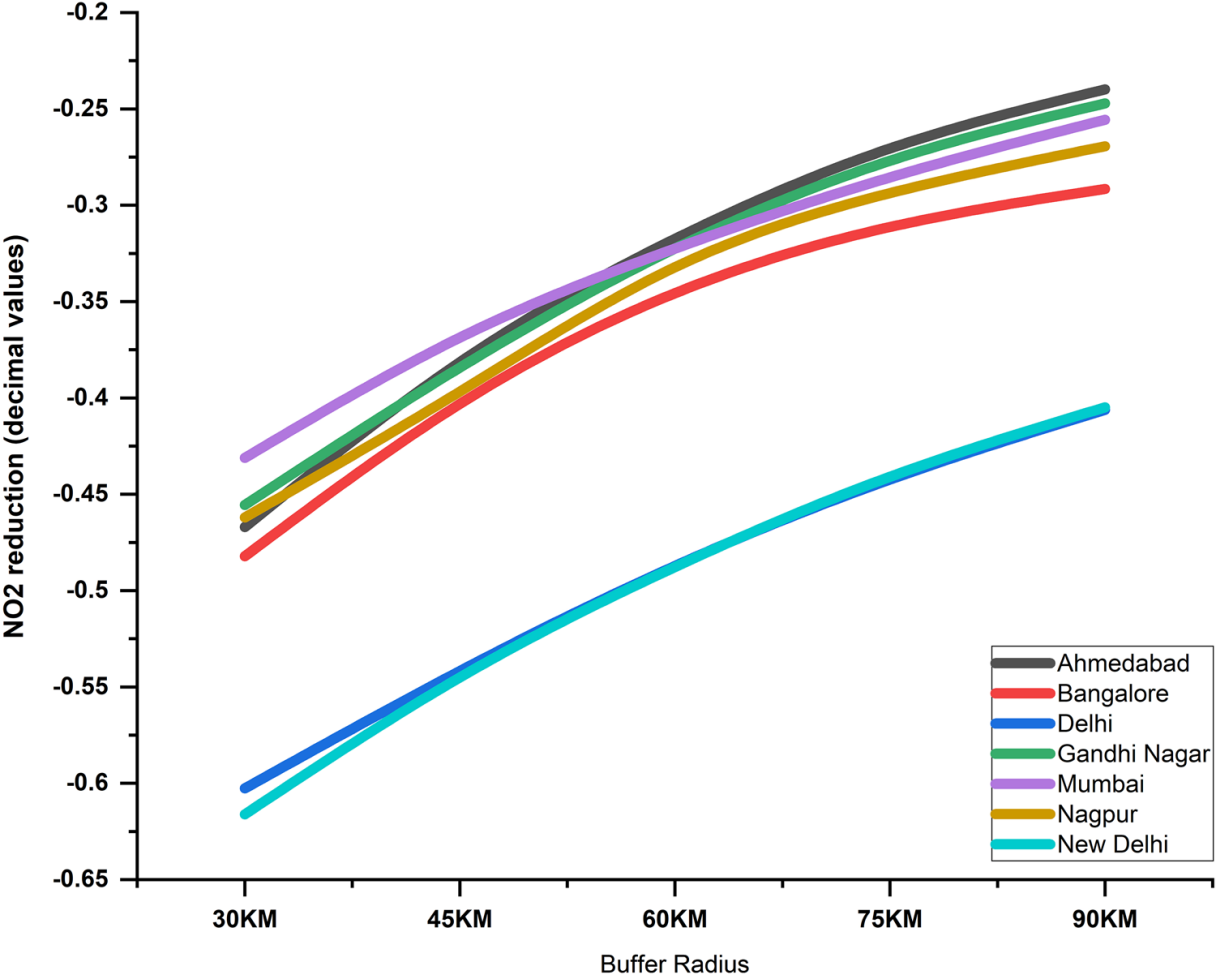
Reduction in tropospheric NO_2_ levels for top seven cities, India with varying buffer distance from the city center during COVID lockdown period (March 25th–May 3rd). As the distance from the city center increased, NO_2_ levels decreased for all seven cities.

We also did a buffer analysis to infer the spatial scaling effects on the reduction in NO2 for different cities. From the city center based on the latitude and longitude, the mean tropospheric NO_2_ were analyzed at varying buffer distances, i.e., 30 km radius, 45, 60, 75, 90 km. Results obtained for NO_2_ reduction (in %) for different cities during the 2020 lockdown period are shown in Fig. 6. Of the different cities, New Delhi and Delhi had the most NO2 reduction (61.6% and 60.2%), followed by Bangalore (48.2%), Ahmedabad (46.70%), Nagpur (46.20%), Gandhi Nagar (45.5%) and Mumbai (43.1%) respectively. Further, for all the cities, as the distance from the city center increased, the NO2 pollution decreased exponentially. Further, of all cities, the highest decrease was noted for Ahmedabad, followed by Gandhi Nagar, Mumbai, etc. (Fig. 6). We attribute the differences to land cover variations within the city including local meteorology impacting pollution in these cities.

#### Time series analysis

**Figure 7:**
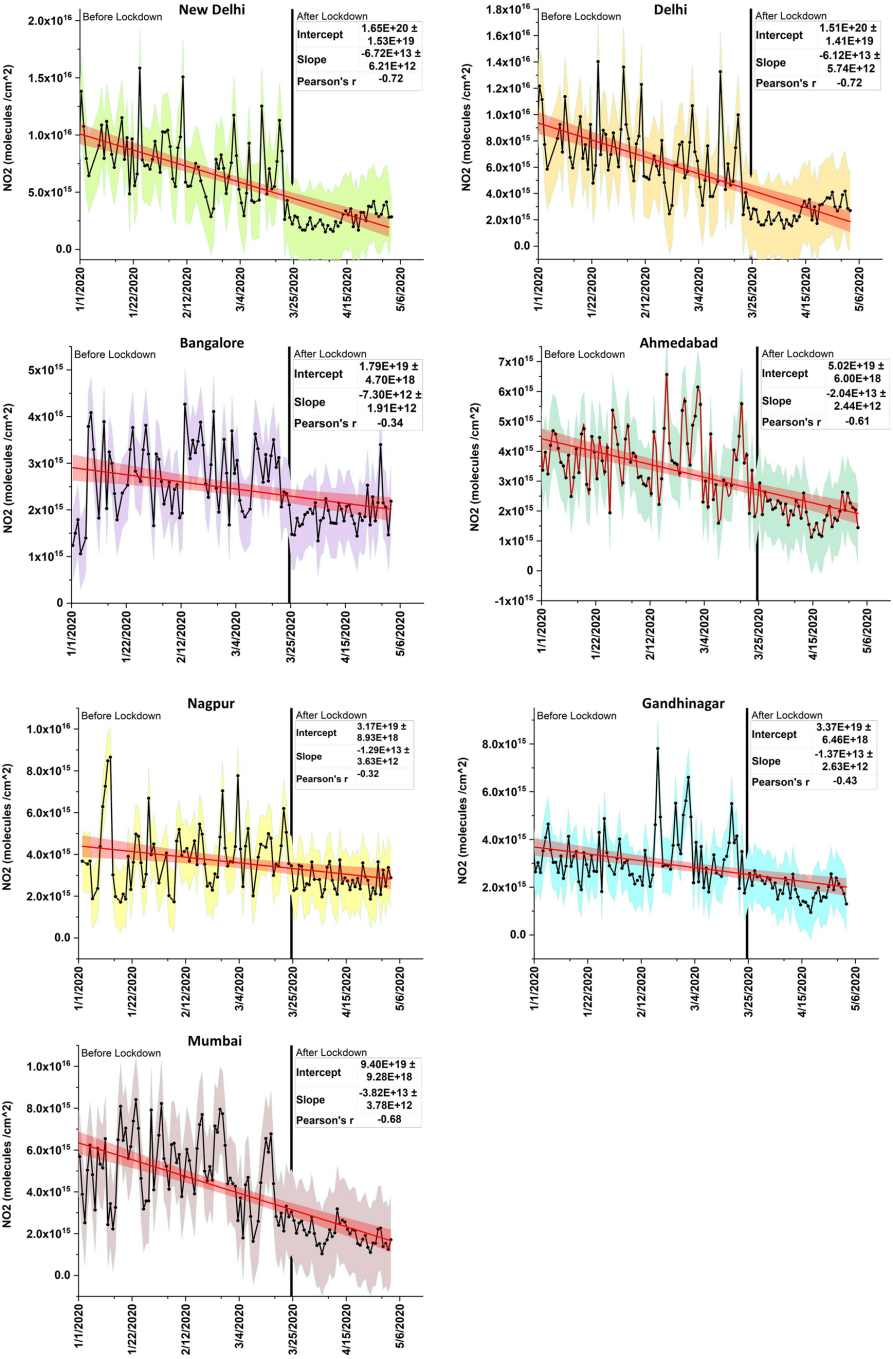
Time series NO_2_ plots for top seven cities, India from January 1 to May 3rd, 2020 COVID. The NO_2_ data is represented as black lines with dots and the standard errors in different shaded colors along with the red regression line and 95% confidence bands in orange. The COVID lockdown period start date (March 3rd, 2020) is shown as vertical black line, after which a clear decline in NO_2_ pollution can be seen for all cities. Trend characteristics of slope, intercept and Pearson’s R for the entire range of data are also given for each plot.

The time series plots for 2020 data from January 1st–May 3rd, 2020, for all the seven cities are shown in Fig. 7. In the figures, data are represented as black lines with the standard errors in the shaded area along with the regression line in red color and 95% confidence bands in orange. In addition, trend characteristics of the slope, intercept, and Pearson’s R is also given for each plot. Thus, for example, both New Delhi and Delhi showed the highest Pearson’s R of − 0.72, followed by Mumbai (− 0.68), Ahmedabad (− 0.61), etc., and least for Nagpur (r = − 0.32). The slope was larger for New Delhi, followed by Delhi, Mumbai, Ahmedabad, etc., and least for Bangalore (Fig. 7).

**Figure 8:**
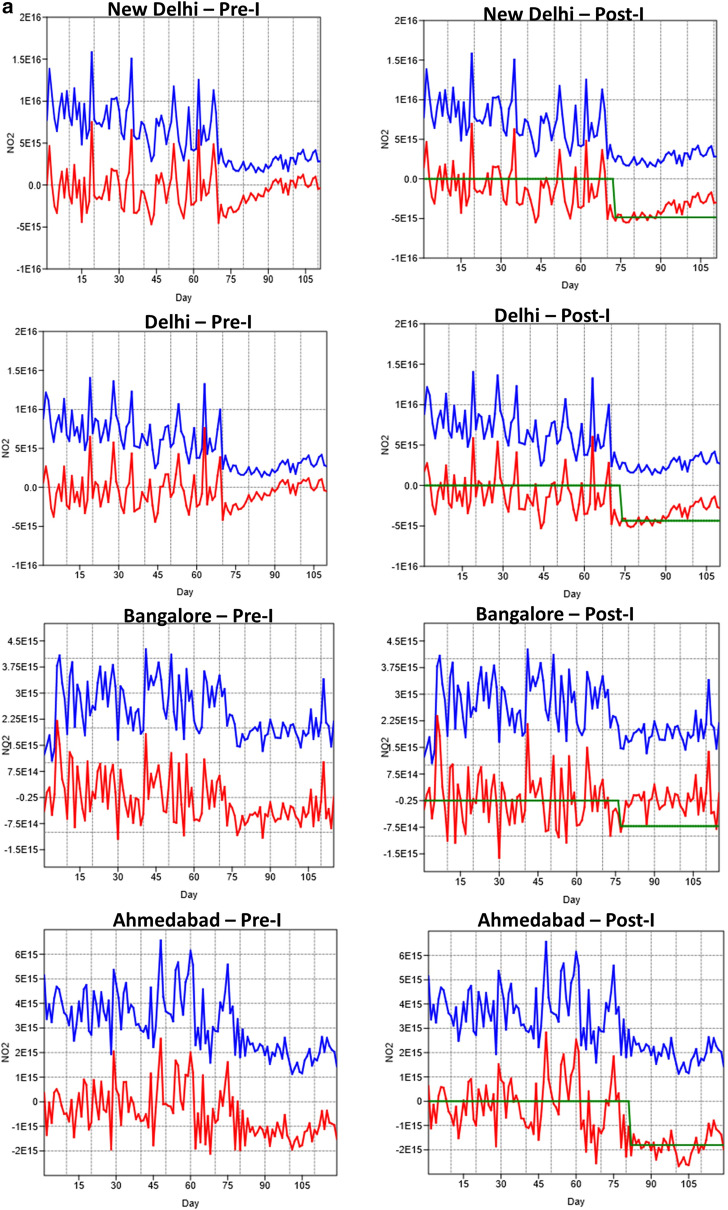

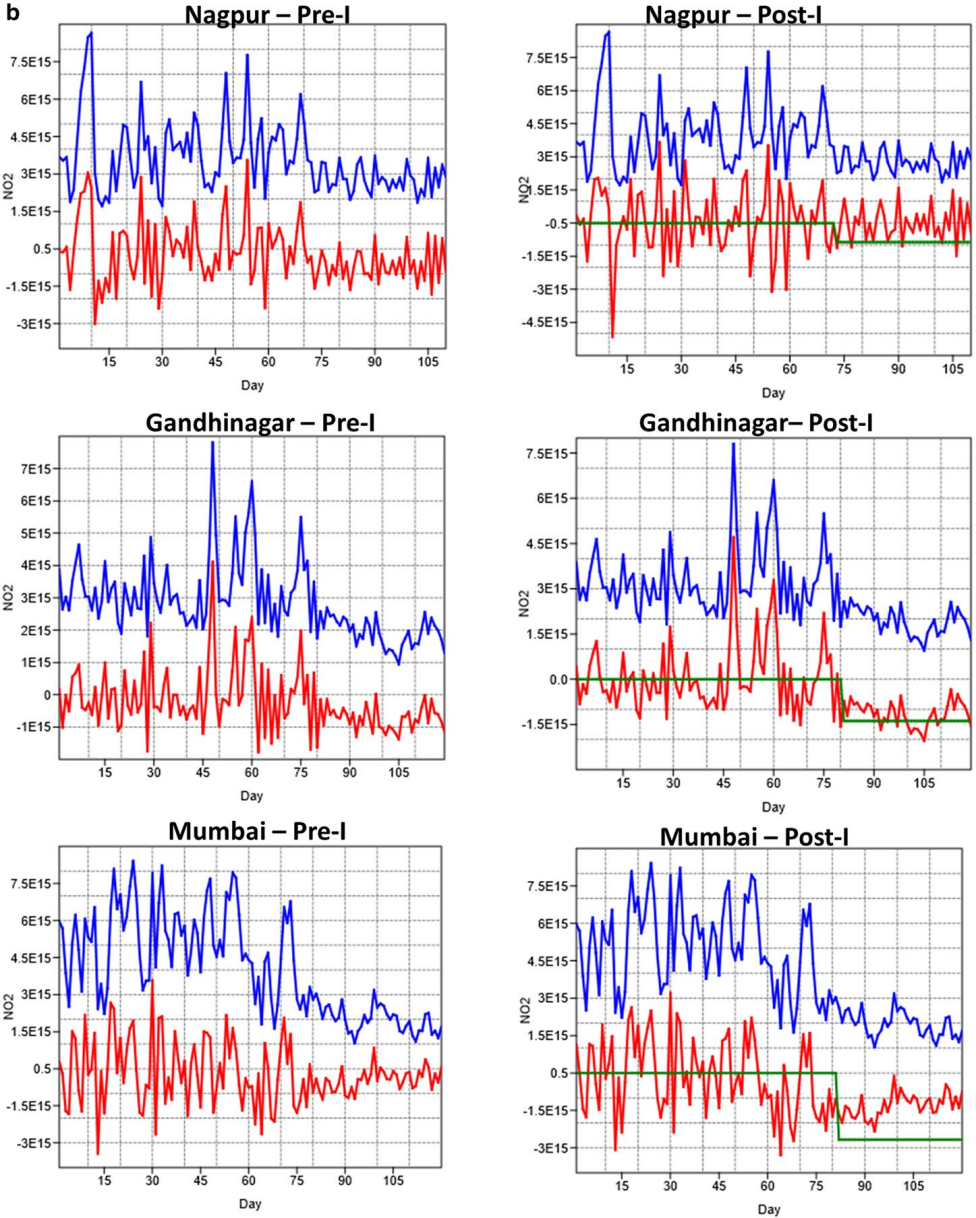
Time series ARMA analysis for the seven cities before (Pre-I) and after (Post-I) COVID intervention. The blue line represents the NO2 data, the redline represents the residuals and the green line represents the intervention. A clear decrease in NO_2_ levels can be seen after day 75 (March 25th, 2020) due to COVID-19.

The ARMA modeling was based on the before COVID (pre-intervention) from January 1, 2020–March 24th, 2020 versus during COVID (post-intervention) from March 25th–May 3rd, 2020. Dividing the entire time series data into two sets helped us to assess the magnitude of differences in NO2 pollution separately. The time series plots for the seven cities before and during COVID intervention is shown in Fig. 8a,b. In the figures, the blue line represents the data, the red line represents the residuals, and the green line represents the intervention. The various AR models fitted for different cities before and after COVID-19 are shown in Tables 1 and 2. An AR(1) autoregressive process is one in which the current value is based on the immediately preceding value, while an AR(2) process is one in which the current value is based on the previous two values. An AR(0) process is used for white noise and has no dependence between the terms. Results suggested a clear decline in NO_2_ pollution (green line in the plots) due to COVID-19. For all cities, the pre-intervention data, too, showed a reduction in NO_2_; however, the reduction was much higher during post-intervention as reflected in the ARMA coefficients. Thus, in all our AR models, the AR coefficients were negative for both pre-and-post intervention COVID data. In particular, for the post-intervention COVID dataset, the coefficients were highly negative and are below 1, suggesting that the NO2 reduction is highly persistent. The size of the moving average window for different cities varied from 0 to 1 for post-intervention COVID data and 1 to 2 for pre-COVID data. Specific to the model performance or measure of goodness of fit, either log-likelihood or AIC can be used. We used both the indicators to assess the consistency in the model performance. The higher the value of Log-likelihood, the better the fit of model coefficients. Thus, for example, post-intervention COVID data consistently had higher values compared to pre-intervention COVID datasets. In contrast to the Log-likelihood estimator, the lesser the AIC value, the better the model performance. Thus, a closer examination of Table (1) AIC values suggests that for most of the post-intervention COVID data, the AIC values are much lower than the pre-intervention COVID data, suggesting higher performance. Both the Log-likelihood and AIC criterion suggested relatively higher model performances for the post-intervention COVID data. Further, for both for the pre-and-post intervention COVID data, both the Log-likelihood and AIC values showed consistency in the order of model performance for different cities. For example, for the pre-intervention COVID data, the Log-likelihood values were higher for Nagpur, followed by New Delhi, Delhi, Bangalore, Gandhinagar and Ahmedabad (Table 2); the AIC followed a similar order with lower values. For the post-intervention COVID data (Table 2), the Log-likelihood ratio estimator showed higher values for Ahmedabad, Delhi, Nagpur, Gandhinagar, Bangalore, Mumbai, and New Delhi and AIC followed a similar order with lower values. The intervention effect can also be assessed in terms of magnitude for both pre-and post-intervention COVID datasets. For both the pre -and post-intervention datasets, the magnitude was negative, suggesting a decrease in pollution as time progressed; however, the values were more negative for the post-intervention COVID data compared to the pre-intervention COVID data. Thus, for the pre-intervention COVID data, a higher reduction in NO_2_ pollution can be seen for New Delhi, followed by Delhi, Ahmedabad, Mumbai, Gandhinagar, Nagpur, and Bangalore. For the post-intervention COVID data, a higher reduction in NO_2_ pollution can be seen for New Delhi, Delhi, Mumbai, Ahmedabad, Gandhinagar, Nagpur, and Bangalore. Further, except for Bangalore, where the NO_2_ reduction was relatively higher for pre-intervention COVID data, for all the other cities, the post-intervention COVID NO_2_ reduction was higher than the pre-intervention COVID datasets. In summary, the ARMA with intervention analysis helped to assess the data in a much more robust way for assessing the pre-and-post intervention COVID related NO_2_ reduction.

**Table 1:** ARMA model parameters before COVID-19 intervention.

Model arameters	New Delhi	Delhi	Bangalore	Ahmedabad	Nagpur	Gandhinagar	Mumbai
AR (coefficients)	1 (− 0.99)	1 (− 0.999)	1 (− 1)	1 (− 0.776)	1 (− 1)	2 (− 1.37; 0.373)	2 (− 1.50; 0.508)
MA (coefficients)	1 (− 0.90)	1 (− 0.88)	2 (− 0.664; − 0.332)	2 (− 0.776; − 0.2246)	2 (− 0.527; − 0.470)	1 (− 0.99)	1 (− 0.938)
Log likelihood	− 2,663	− 2,695	− 2,711	− 2,916	− 2,614	− 2,883	− 2,945
AIC	5,329	5,393	5,428	5,838	5,234	5,772	5,896
Magnitude	− 3.73E15	− 2.19E15	− 8.27E14	− 1.8E15	− 1.26E15	− 1.322E15	− 1.732E15
Standard error	1.67E15	1.716E15	− 2.872E14	3.76E14	5.96E14	4.872E14	1.38E15

**Table 2:** ARMA model parameters after COVID-19 intervention.

Model parameters	New Delhi	Delhi	Bangalore	Ahmedabad	Nagpur	Gandhinagar	Mumbai
AR (coefficients)	1 (− 1)	1 (− 1)	1 (− 0.99)	3 (− 0.911, 0.063; − 0.152)	1 (− 0.918)	1 (− 1)	1 (− 0.99)
MA (coefficients)	1 (− 0.998)	1 (0.999)	1 (0.637)	1 (− 0.996)	0	1 (− 0.998)	1 (− 0.595; − 0.370)
Log likelihood	− 1,442	− 1,366	− 1,395	− 1,360	− 1,386	− 1,392	− 1,423
AIC	2,888	2,735	2,793	2,727	2,774	2,787	2,851
Magnitude	− 4.85E15	− 4.36E15	− 7.18E14	− 1.803E15	− 8.64E14	− 1.38E15	− 2.67E15
Standard error	8.061E14	7.99E14	8.84E14	2.76E14	2.116E15	3.34E14	7.54E14

### NO_2_–MODIS-AOD relationships

We also explored whether the MODIS AOD could capture variations in pollution reduction. Although the spatial patterns in tropospheric NO_2_ and MODIS AOD matched (Supplementary File), they were poorly correlated. For example, the Pearson correlation coefficient (r) for New Delhi was (0.128), Delhi (0.11), Bangalore (0.02), Ahmedabad (0.07), Nagpur (0.08), Gandhinagar (0.03) and Mumbai (0.09). The poor correlations can be attributed to the inherent nature of the data. For example, the MODIS AOD datasets represent coarse and fine particulate aerosols (including dust) for the entire column of the atmosphere, whereas the NO_2_ data represents the data for only the troposphere. Despite these differences, both the datasets showed overall decreasing mean concentrations during the 2020 lockdown period, and the temporal patterns matched for the specific dates.

### Variations in NO_2_ based on the population rank

Results from the 2019 no-lockdown period versus 2020 lockdown period for various cities based on the population ranks are shown in Table 3. Various population rank categories are as follows: Rank-1 (5.0 million and greater); Rank-2 (1.0 to 4.9 million); Rank-3 (500,000–999,999); Rank-4 (250,000–499,000); Rank-5 (100,000–249,000); Rank-6 (500,00–99,999); Rank-7 (< 50,000). Thus, for Rank-1 population cities, the mean reduction in NO_2_ was 51%, Rank-2—30%, etc. In the case of Rank-5 and Rank-6 cities, there was an increase in pollution of 3% and 22%, respectively. We attribute the differences to the geographical location; for example, most of the cities (not all) in these two ranks are coastal with dominant wind and sea breeze influences, compared to the other cities.

**Table 3:**
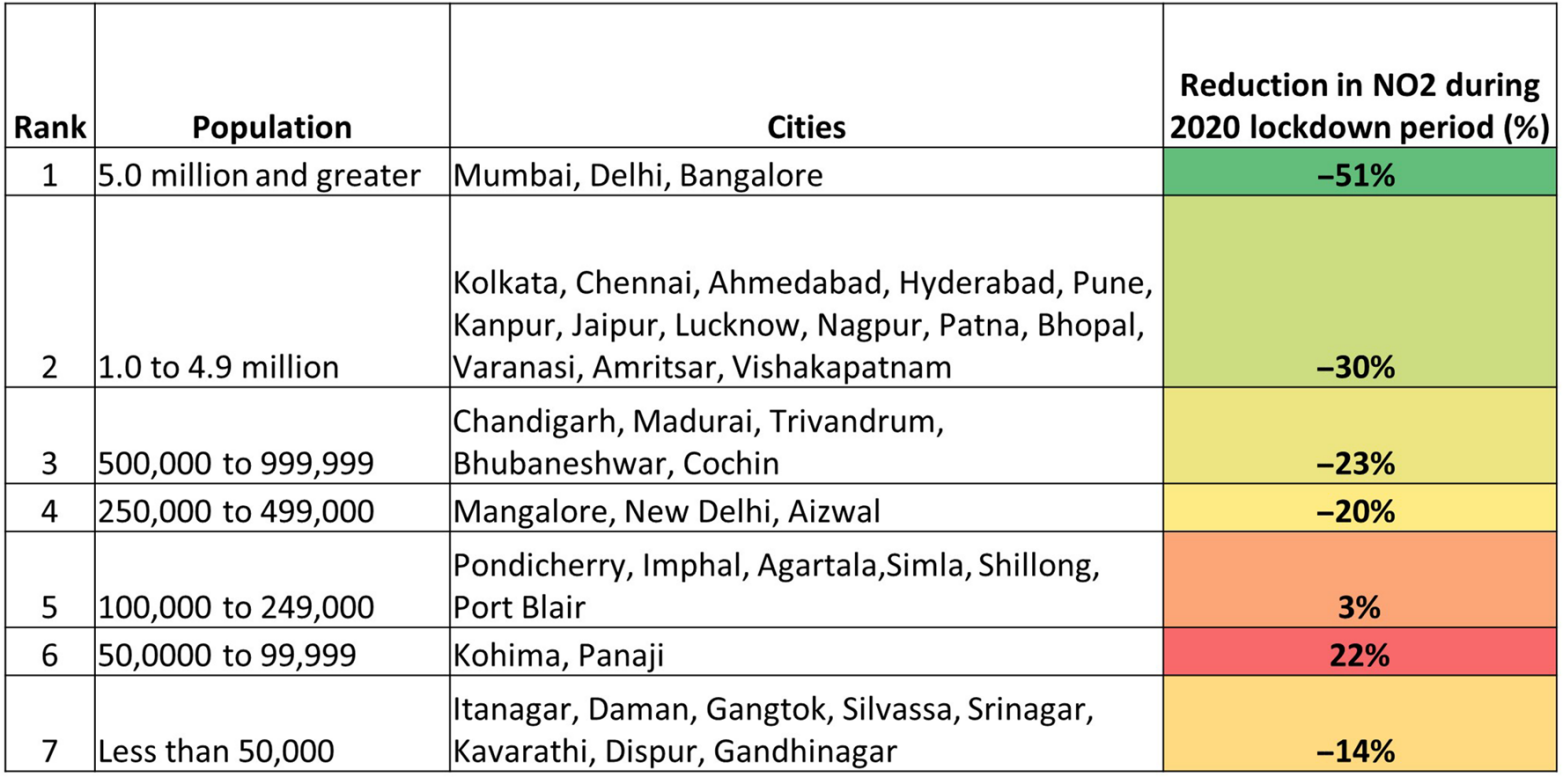
NO_2_ reduction (%) aggregated based on population ranks for 41 different cities of India.

Except for rank-5 and 6 cities studied, there was a reduction in NO_2_ levels during 2020 lockdown period (March 25th to May 3rd) compared to 2019 similar dates.

See Supplementary Material for details on individual cities.

### Variations in NO_2_ in northeast Indian cities

**Figure 9:**
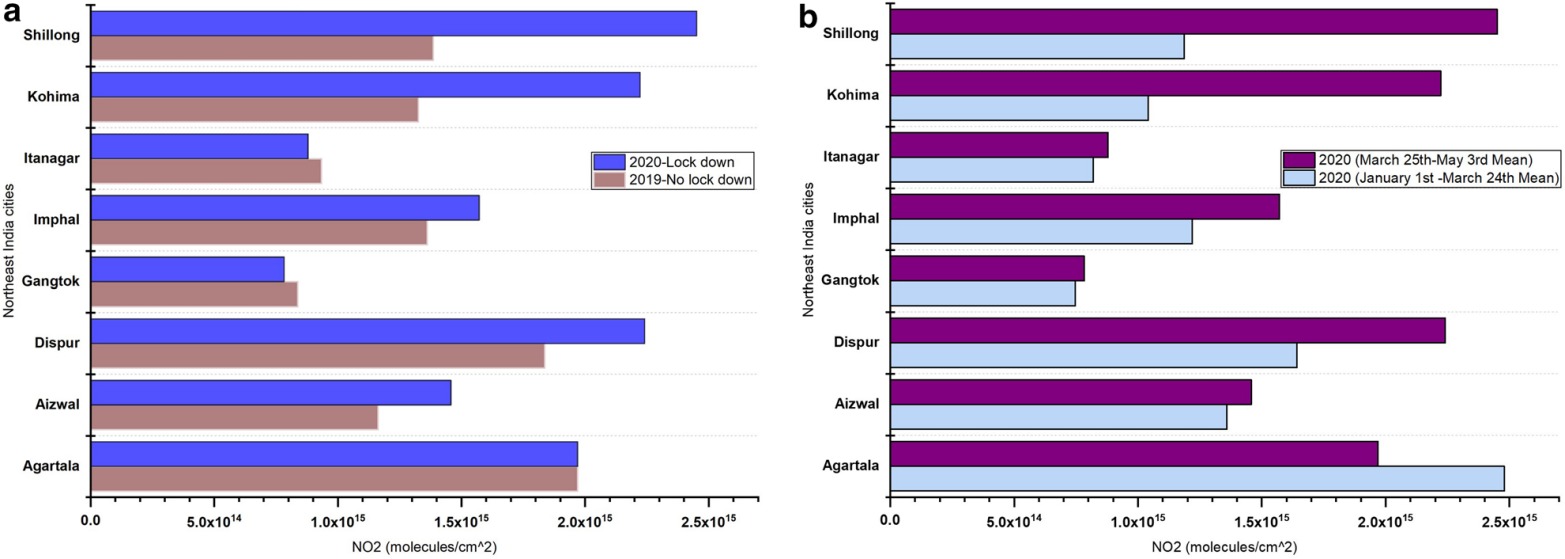
(**a**). Variations in NO_2_ for Northeast India cities in India during 2019 no lock down period (March 25th–May 3rd) and 2020 lock down period (March 25th–May 3rd); (**b**)**.** Data shown for 2020 no lock down period (averaged NO_2_ data from January 1st to March 24th) and 2020 lock down period (March 25th–May 3rd).

**Figure 10:**
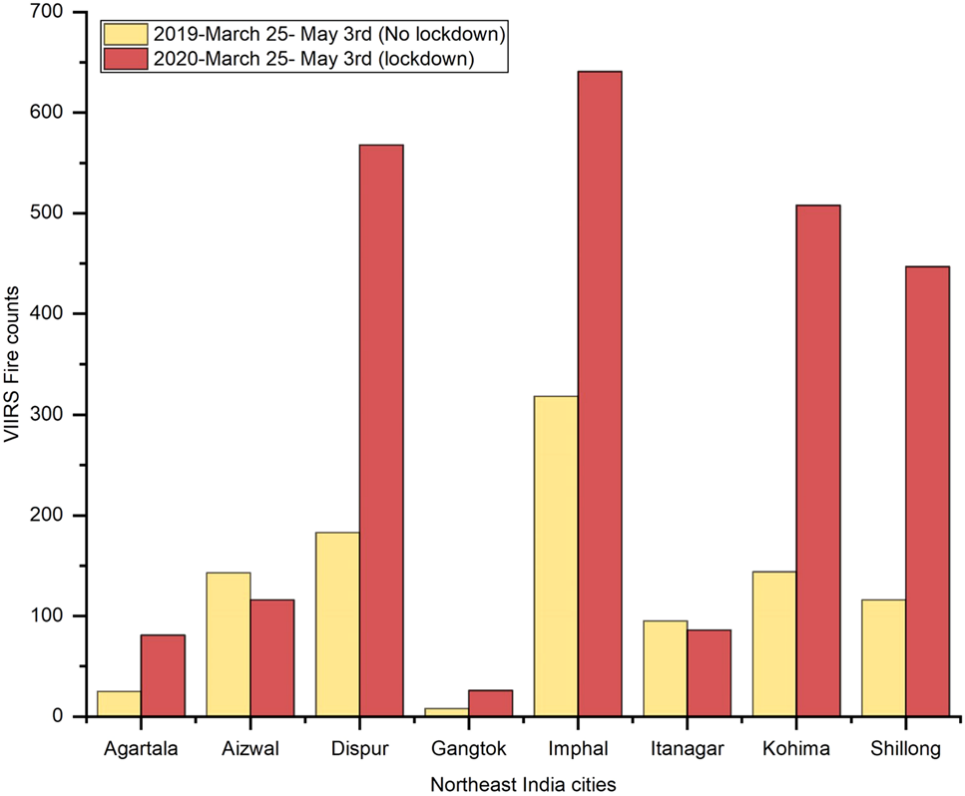
Variations in vegetation fires in Northeast India during 2019 no lockdown and 2020 lockdown period. A clear increase in vegetation fires can be seen for five different cities during 2020 which resulted in an increase in NO_2_ levels during the COVID lockdown period. The specific dates are shown on the top of the Figure.

In contrast to other cities, northeast Indian cities had an almost 24% increase in NO_2_ levels during the 2020 lockdown period compared to the 2019 no-lockdown period during similar dates. Also, a comparison of NO_2_ levels for 2020 pre-lockdown versus post lockdown suggested an average NO_2_ increase of 36% during the 2020 lockdown period for the cities in northeast India (Fig. 9a,b). Our preliminary analysis of VIIRS active fire data suggests that an increase in NO_2_ levels may be due to vegetation fires, which increased during the 2020 lockdown period compared to 2019 during non-lockdown periods, especially in areas around the cities of Imphal, Dispur, Kohima, Shillong and Agartala (Fig. 10). A detailed analysis of NO_2_ increase in relation to vegetation fires using daily datasets is ongoing.

### Variations in NO_2_ in coastal cities

**Figure 11:**
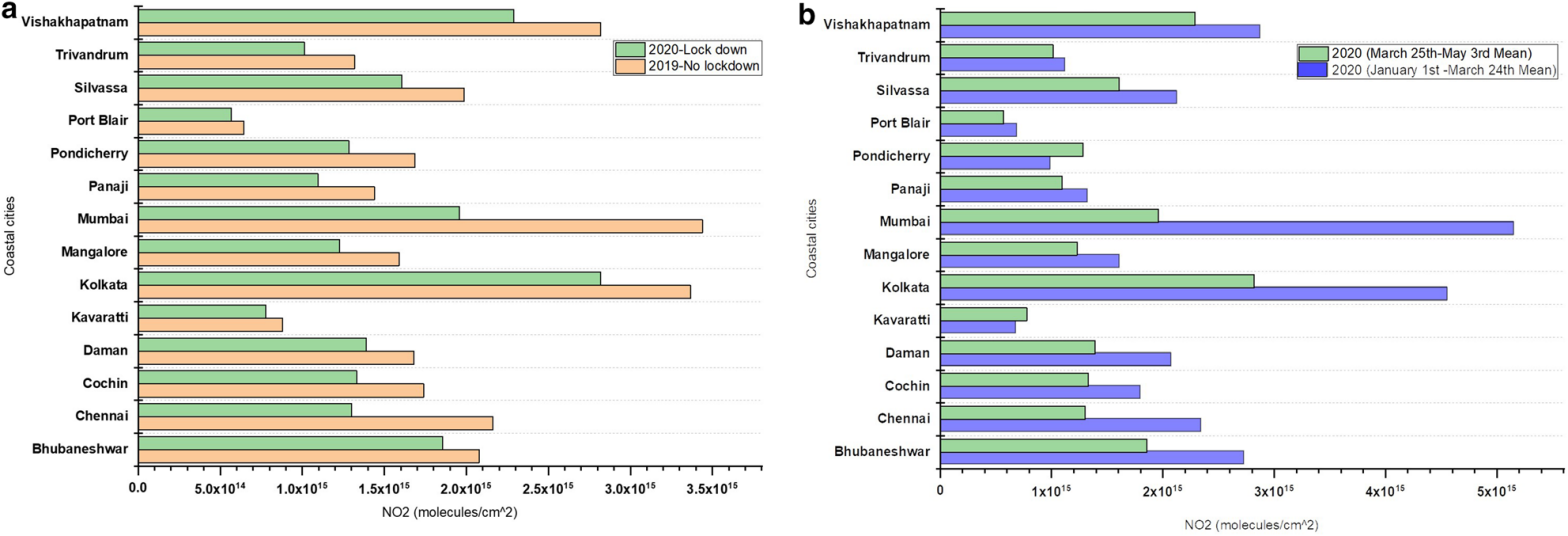
(**a**). Variations in NO_2_ for Coastal cities in India during 2019 no lock down period (March 25th–May 3rd) and 2020 lock down period (March 25th–May 3rd); (**b**). Data shown for 2020 no lock down period (averaged NO_2_ data from January 1st to March 24th) and 2020 lock down period (March 25th–May 3rd).

Coastal cities had almost 22% NO_2_ reduction during the 2020 lockdown period compared to the 2019 no-lockdown period with the highest in Mumbai and Kolkata (Fig. 11a). Also, a comparison of NO_2_ levels for 2020 pre-lockdown versus post lockdown suggested an average NO_2_ reduction of 30% during the 2020 lockdown period with the highest reduction in Mumbai with a 43.08% reduction (Figs. 5 and 11b). However, the overall NO_2_ reduction during the 2020 lockdown period is relatively lower for the coastal cities compared to the top six non-coastal cities of New Delhi (61.74%), Delhi (60.37%), Bangalore (48.25%), Ahmedabad (46.20%), Gandhinagar (45.64%), and Nagpur (46.13%).

### Ground-based measurements

We obtained the ground-based NO_2_ measurement data (µg/m^3^) for fifteen different cities of the total 41 cities of our current focus from the Central Pollution Control Board (CPCB)^24^, India. Additional data for the other cities that matched our currently studied cities including spatial and temporal data from the CPCB were not available. The mean monthly NO_2_ values during the April lockdown period for different cities from the ground stations are as follows: Ahmedabad (16.24), Aizawl (0.532), Bangalore (7.934), Bhopal (11.06), Chandigarh (12.63), Chennai (4.53), Delhi (24.17), Gandhinagar (2.506), Hyderabad (23.54), Jaipur (12.34), Kanpur (20.78), Kolkata (15.61), Lucknow (19.93), Mumbai (4.43), Nagpur (19.42), Varanasi (31.68). Further, a comparison of the March 2020 values for these cities suggested an 18% reduction due to COVID-19 lockdown. These results also match closely with the reduction in NO_2_ reported for some of the cities using ground- based measurements^6,7^. Also, correlating the TROPOMI tropospheric NO_2_ data for the April lockdown period suggested a Pearson (r) of 0.33. The poor correlation can be attributed to the satellite data resolution aspects (3.5 × 7 km^2^), compared to the ground station data footprint which might be much smaller than the satellite footprint. In addition, we infer that more ground station data at both spatial and temporal scales is required to validate the satellite data.

## Discussion and conclusion

An overview of the results suggests significant differences and patterns in NO_2_ and AOD which are briefly highlighted. India has four climatological seasons^25^, Winter (December–February), Summer or Pre-monsoon (March–May), Monsoon or rainy season (June to September) and Post-monsoon or autumn season (October–November). Thus, the 2020 lockdown period mostly occurred during the Summer or pre-monsoon season. In general, most of the cities in the northern part of India see elevated pollution during the post-monsoon season due to the combined effect of anthropogenic and atmospheric factors. For example, in states of Punjab and Haryana, important sources of pollution include agricultural residue burning, industrial and vehicular emissions, dust storms, burning of solid fuels for heating, etc., which cause elevated pollution levels not only in these states but also the neighboring capital city, New Delhi^26^. In addition, during the post-monsoon season, due to the temperature inversion, there is less dispersion of pollutants resulting in smog events. In contrast, during the summer, the dispersion of pollutants is relatively higher compared to the post-monsoon season; the warmer air is lighter and rises upwards more easily carrying the pollutants away from the land surface and mixes the pollutants with the clear air in the upper layers of the atmosphere^27,28^, resulting in lesser concentrations. In addition to the summer effect, due to the COVID-19 lockdown, we found a significant reduction in pollution in major metropolitan cities. We found several variations, with some cities having more reduction in NO_2_ than others in northeast India which experienced an increase in pollution due to the fires during the COVID-19 lockdown period. More thorough research is needed to understand the fire phenomenon, emissions and meteorology using the daily datasets. Also, in the coastal areas, the impact of sea and bay breezes on air quality, including air-mass transportation studies needs to be examined to address the spatial and temporal variations. We also infer the need to validate satellite measurements with the ground-based measurements. Our results on NO_2_ reduction during the COVID-19 lockdown period match with the other studies conducted for some of the cities using the ground-based measurements and CPCB data from India.

Overall, this study focused on COVID-19 impacts on NO_2_ pollution. Our results suggested a significant reduction in NO_2_ during the lockdown period for most of the cities of India, except those located in Northeast India. The results from the study include variation in NO_2_ based on geographical location, population ranks, distance from the city center, and robust statistical tests to determine the significance of a change in 41 different cities. Interestingly, we found notably higher vegetation fires during the lockdown period in Northeast Indian cities which warrant further investigation. The adverse effects of NO_2_ pollution are well known in the literature. For example, higher doses of NO_2_ can cause respiratory ailments^8^. Also, NO_2_ and other oxides of nitrogen can react with water, oxygen and other chemicals to form acid rain which can be harmful to fish and other wildlife. The acid rain can washout nutrients and minerals from the soil damaging the crops and vegetation including damage to buildings and structures. Considering these detrimental effects, it is important to arrive at effective NO_2_ pollution abatement strategies. Specific to the pollution abatement, the issue of spatial scale is increasingly being realized. Our results reveal greater variations in terms of NO_2_ with some cities having the highest reduction compared to the other based on location and also variations based on the distance to the city center. Thus, policies to mitigate air pollution can be framed based on the local pollutant variations, needs and priorities. The spatial NO_2_ variations highlighted in 41-different cities in our study can serve as a benchmark to address such variations and can help decision-makers to arrive at efficient air quality management plans involving local stakeholders. Although a temporary lockdown in emissions due to COVID-19 is a minor reduction in the overall pollution footprint, the current situation provides some useful insights on how policies like mandatory lockdown can have a measurable positive impact on the pollution control. As the economy reopens, the emissions will rebound, however, some of the policies such as working remotely could keep emissions under control post-COVID-19 situation. We also infer a strong need for a political will and social interventions to curb pollution beyond COVID-19 in India.

## Supplementary information

Supplementary information.
