# Supplementary material for: Spatial and temporal variations of air pollution over 41 cities of India during the COVID-19 lockdown period
Source: Sci Rep. 2020 Oct 6;10:16574. doi: 10.1038/s41598-020-72271-5 (PMC7539013; doi:10.1038/s41598-020-72271-5)
Supplement: Supplementary file 1 — Supplementary information. [file 41598_2020_72271_MOESM1_ESM.pdf]

Spatial and temporal variations of Air pollution over 41 cities of India during the COVID-19 lockdown period

Krishna Prasad Vadrevu[1]*, Aditya Eaturu[2], Sumalika Biswas[3], Kristofer Lasko[4], Saroj Sahu[5], Garg JK[6] and Chris Justice[7]

[1]NASA Marshall Space Flight Center, Huntsville, Alabama, USA
[2]University of Alabama in Huntsville, USA
[3]Smithsonian Conservation Biology Institute, Virginia, USA
[4]US Army Corps of Engineers, Alexandria, Virginia, USA
[5]Utkal University, Bhubaneswar, Odisha, India
[6]Tata Energy Research Institute (TERI) School of Advanced Studies, New Delhi, India
[7]University of Maryland, College Park, Maryland, USA

*Corresponding Author

Krishna Prasad Vadrevu[1*], NASA Marshall Space Flight Center, Huntsville, Alabama, USA, 35811; Email: krishna.p.vadrevu@nasa.gov

Supplementary Table and Figure enclosed (next page)

CITY NAME	X	Y	2019-March 25th to May 3rd Mean NO2	2020-March 25th to May 3rd Mean NO2	Difference %
Agartala	91.26670	23.81670	1.969E+15	1.969E+15	0%
Ahmadabad	72.56687	23.03979	3.692E+15	1.969E+15	-47%
Aizawl	92.73330	23.66670	1.162E+15	1.457E+15	25%
Amritsar	74.87156	31.63089	2.270E+15	1.560E+15	-31%
Bangalore	77.58773	12.97475	3.619E+15	1.873E+15	-48%
Bhopal	77.41200	23.23000	2.156E+15	1.813E+15	-16%
Bhubaneshwar	85.83500	20.26400	2.078E+15	1.855E+15	-11%
Chandigarh	76.78400	30.73800	2.614E+15	1.728E+15	-34%
Chennai	80.24800	13.06200	2.162E+15	1.301E+15	-40%
Cochin	76.28042	9.97016	1.740E+15	1.331E+15	-24%
Daman	72.85000	20.41637	1.680E+15	1.391E+15	-17%
Delhi	77.21666	28.66667	6.504E+15	2.577E+15	-60%
Dispur	91.78330	26.13330	1.837E+15	2.240E+15	22%
Gandhinagar	72.66667	23.20000	3.523E+15	1.915E+15	-46%
Gangtok	88.61700	27.32900	8.371E+14	7.829E+14	-6%
Hyderabad	78.48500	17.39500	3.740E+15	2.306E+15	-38%
Imphal	93.94100	24.78800	1.361E+15	1.572E+15	15%
Itanagar	93.55000	27.15000	9.334E+14	8.792E+14	-6%
Jaipur	75.80100	26.90500	2.746E+15	1.885E+15	-31%
Kanpur	80.31786	26.45783	2.565E+15	1.957E+15	-24%
Kavaratti	72.63315	10.55095	8.792E+14	7.769E+14	-12%
Kohima	94.11600	25.66800	1.325E+15	2.222E+15	68%
Kolkata	88.33400	22.54400	3.366E+15	2.818E+15	-16%
Lucknow	80.92000	26.84900	2.704E+15	1.939E+15	-28%
Madurai	78.12173	9.91399	1.885E+15	1.421E+15	-25%
Mangalore	74.84300	12.86900	1.590E+15	1.229E+15	-23%
Mumbai	72.82600	19.07700	3.439E+15	1.957E+15	-43%
Nagpur	79.08911	21.15575	5.131E+15	2.764E+15	-46%
New Delhi	77.21675	28.56873	6.925E+15	2.650E+15	-62%
Panaji	73.81800	15.49200	1.439E+15	1.096E+15	-24%
Patna	85.13500	25.61400	2.830E+15	2.114E+15	-25%
Pondicherry	79.83700	11.93300	1.686E+15	1.283E+15	-24%
Port Blair	92.75833	11.66756	6.444E+14	5.667E+14	-12%
Pune	73.85227	18.53574	3.174E+15	2.102E+15	-34%
Shillong	91.88333	25.56666	1.385E+15	2.451E+15	77%
Silvassa	73.00179	20.19155	1.987E+15	1.608E+15	-19%
Simla	77.16600	31.10400	2.005E+15	1.229E+15	-39%
Srinagar	74.80400	34.08100	1.608E+15	1.144E+15	-29%
Trivandrum	76.95200	8.50400	1.319E+15	1.012E+15	-23%
Varanasi	82.95634	25.28201	3.125E+15	2.439E+15	-22%
Vishakhapatnam	83.33000	17.75000	2.818E+15	2.288E+15	-19%
**Mean**					-19%

Table. NO2 variations in 41 different cities during 2019-non lockdown and 2020 (lockdown period)

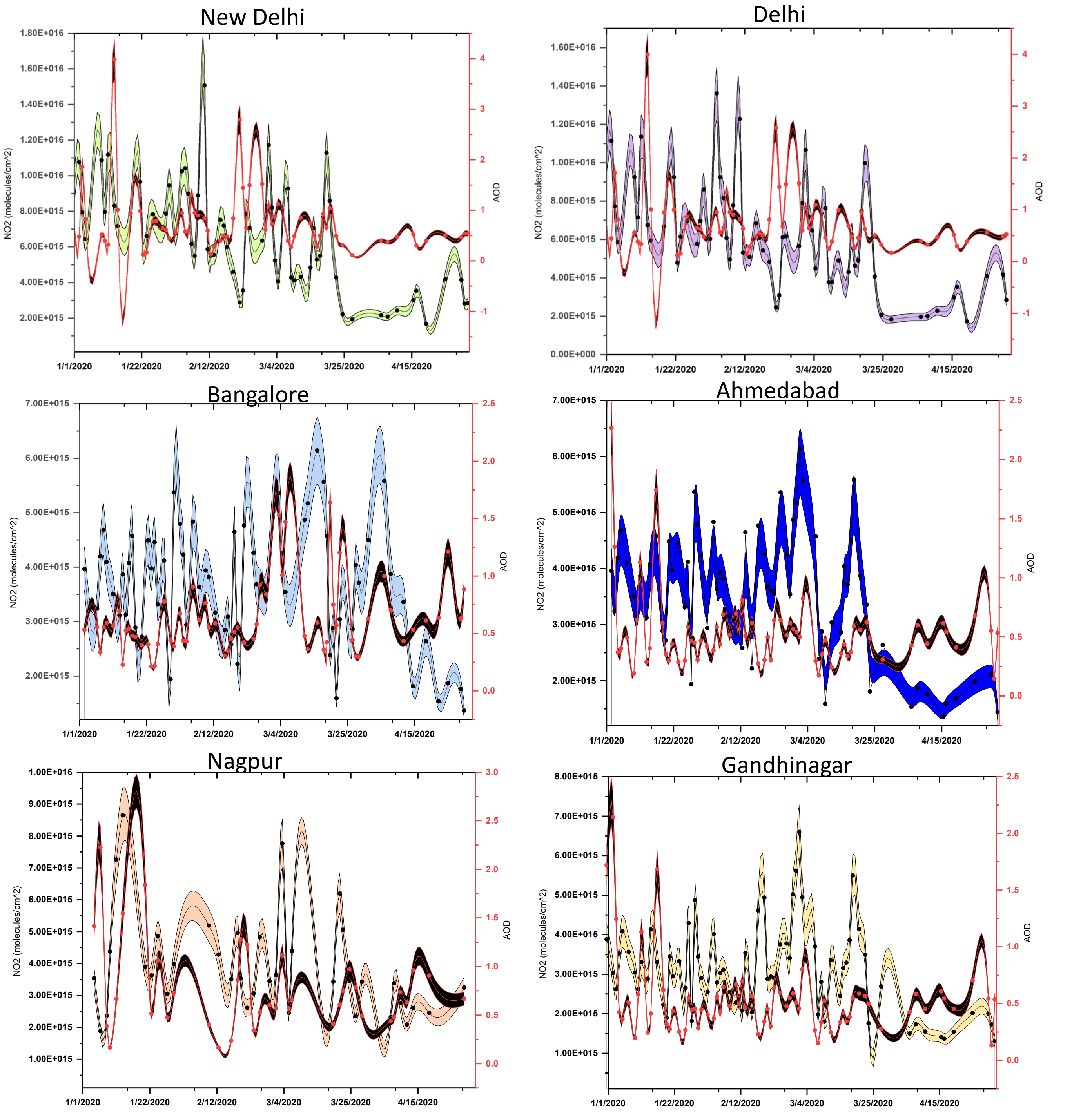

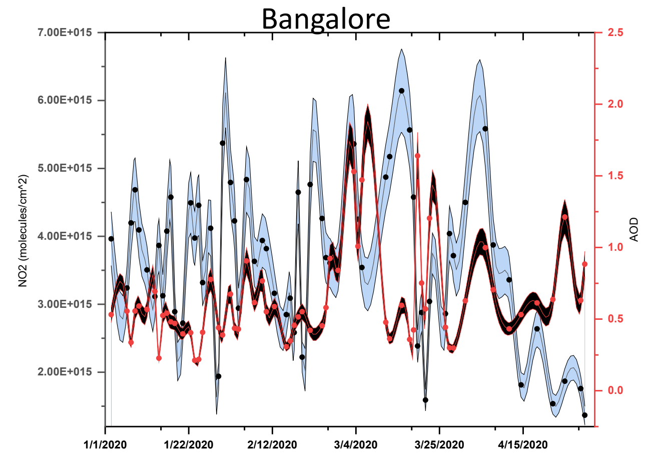

Figure. Time series plots of NO_2 and MODIS datasets with NO_2 data in different colors and MODIS AOD in red line, both with standard errors in different shaded areas. Although the NO_2-MODIS correlations were not strong, an overall decrease in NO2 and AOD were observed for different cities during 2020 lockdown period due to COVID-19.
